# Transcriptome and Proteome of Fish-Pathogenic *Streptococcus agalactiae* Are Modulated by Temperature

**DOI:** 10.3389/fmicb.2018.02639

**Published:** 2018-11-02

**Authors:** Guilherme C. Tavares, Alex F. Carvalho, Felipe L. Pereira, Cristiana P. Rezende, Vasco A. C. Azevedo, Carlos A. G. Leal, Henrique C. P. Figueiredo

**Affiliations:** ^1^AQUACEN–National Reference Laboratory of Aquatic Animal Diseases, Ministry of Agriculture, Livestock and Food Supply, Veterinary School, Federal University of Minas Gerais, Belo Horizonte, Brazil; ^2^LGCM–Laboratory of Cellular and Molecular Genetics, Biological Science Institute, Federal University of Minas Gerais, Belo Horizonte, Brazil

**Keywords:** GBS, temperature, fish, microarray, label-free shotgun proteome

## Abstract

*Streptococcus agalactiae* is one of the most important pathogens associated with streptococcosis outbreaks in Nile tilapia farms worldwide. High water temperature (above 27°C) has been described as a predisposing factor for the disease in fish. At low temperatures (below 25°C), fish mortalities are not usually observed in farms. Temperature variation can modulate the expression of genes and proteins involved in metabolism, adaptation, and bacterial pathogenicity, thus increasing or decreasing the ability to infect the host. This study aimed to evaluate the transcriptome and proteome of a fish-pathogenic *S. agalactiae* strain SA53 subjected to *in vitro* growth at different temperatures using a microarray and label-free shotgun LC-HDMS^E^ approach. Biological triplicates of isolates were cultured in BHIT broth at 22 or 32°C for RNA and protein isolation and submitted for transcriptomic and proteomic analyses. In total, 1,730 transcripts were identified in SA53, with 107 genes being differentially expressed between the temperatures evaluated. A higher number of genes related to metabolism, mainly from the phosphotransferase system (PTS) and ATP-binding cassette (ABC) transport system, were upregulated at 32°C. In the proteome analysis, 1,046 proteins were identified in SA53, of which 81 were differentially regulated between 22 and 32°C. Proteins involved in defense mechanisms, lipid transport and metabolism, and nucleotide transport and metabolism were upregulated at 32°C. A higher number of interactions were observed in proteins involved in nucleotide transport and metabolism. We observed a low correlation between the transcriptome and proteome datasets. Our study indicates that the transcriptome and proteome of a fish-adapted *S. agalactiae* strain are modulated by temperature, particularly showing differential expression of genes/proteins involved in metabolism, virulence factors, and adaptation.

## Introduction

*Streptococcus agalactiae* (Lancefield's Group B *Streptococcus*, GBS) is one of the most important pathogens associated with disease outbreaks in farm-raised Nile tilapia in Brazil (Salvador et al., 2005; Mian et al., 2009; Chideroli et al., 2017), being responsible for significant economic losses annually (Mian et al., 2009). The pathogen causes septicemia and meningoencephalitis in different fish species from freshwater, estuarine, and marine environments worldwide (Evans et al., 2002), and commonly affects adult fish (Mian et al., 2009).

High water temperature, intensive husbandry, and high stock densities are considered as risk factors for streptococcal infection in tilapia (Zamri-Saad et al., 2014). A higher number of GBS outbreaks has been observed during the summer season when the water temperature is >27°C and a higher thermal amplitude is observed during the day (Mian et al., 2009; Kayansamruaj et al., 2014). Fish mortalities resulting from GBS infection are not usually observed when the water temperature is below 25°C (Rodkhum et al., 2011; Marcusso et al., 2015; Chideroli et al., 2017).

In an aquatic environment, fish are often exposed to spatial and temporal variations in temperature that affect the physiological traits and survival of the aquatic host (Boltaña et al., 2017). In addition, variation in water temperature can influence the fish immune response against bacterial infection as well as modify the morphology, metabolism, and pathogenicity of bacteria by increasing or decreasing the host susceptibility to infection (Mereghetti et al., 2008; Kayansamruaj et al., 2014; Zhao et al., 2015).

In this context, global changes in gene and protein expression resulting from adaptation to a particular niche in the host or environment can be evaluated through transcriptomic and proteomic approaches using different methods (Tian et al., 2013; Silva et al., 2014). The microarray approach allows analysis of global gene expression in a microorganism under a given experimental condition (Tian et al., 2013). In contrast, proteomic studies using liquid chromatography-mass spectrometry (LC-MS) allow evaluation of the global expression of the functional genome in a bacterial pathogen at the protein level, under given experimental conditions (Silva et al., 2014).

Regarding thermal stress, small temperature variations represent a challenge to pathogen survival. These effects can block the cell cycle, culminating in stagnation of bacterial growth and proliferation, or depending on the severity of heat stress, cause bacterial death (Richter et al., 2010). Cold shock proteins can alter the bacterial membrane fatty acid composition and global protein profile to prevent cold-induced decreases in membrane fluidity, reduced protein synthesis, inefficient protein folding, and changes in nucleic acid structures (Phadtare and Severinov, 2010). All microorganisms respond to temperature variation through increased expression of heat shock or cold shock proteins, which act as activators or repressors of several other proteins (Ehira et al., 2009). Evaluation of the temperature-induced transcriptome using microarray technology has been reported in *Yersinia pestis* (Han et al., 2004), *Escherichia coli* (Gadgil et al., 2005), *Streptococcus pyogenes* (Smoot et al., 2001), and GBS (Mereghetti et al., 2008), whereas shotgun proteomics analysis has been used to evaluate differential protein abundance induced by temperature in *E. coli* (Kocharunchitt et al., 2012), *Ochrobactrum anthropi* (Varano et al., 2016), and *Bacillus weihenstephanensis* (Stelder et al., 2015).

A previous study has demonstrated that when cultivated at 35°C, GBS shows raid growth, higher hemolytic activity, and a higher viability in tilapia whole blood compared to cultivation at 28°C. In addition, several virulence genes were upregulated at this temperature, inducing a higher mortality rate in infected fish (Kayansamruaj et al., 2014). However, there are no studies demonstrating the global expression of the functional genome of fish-adapted GBS strains at the transcript and protein levels under low or high temperature conditions.

Thus, this study aimed to evaluate the transcriptome and proteome of *Streptococcus agalactiae*, isolated from diseased fish and subjected to *in vitro* growth at different temperatures, using microarray and liquid chromatography-mass spectrometry label-free shotgun (LC-HDMS^E^) approaches.

## Materials and methods

### Bacterial strains and growth conditions

*S. agalactiae* SA53 (ST-260) isolated from diseased fish was used in this study. The strain was selected from a culture collection at the National Reference Laboratory for Aquatic Animal Diseases (AQUACEN), and belongs to the most frequent genotype occurring in Latin America (Evans et al., 2008; Godoy et al., 2013; Barato et al., 2015). The complete genome sequence is available in GenBank (accession number CP019802.1; Barony et al., 2017). The strain was streaked on 5% sheep blood agar and incubated at 28°C for 48 h. Colonies were then inoculated in triplicate cultures of 100 mL BHI broth (Brain Heart Infusion broth, Himedia, Mumbai, India) with 0.05% (v/v) Tween 80 (BHIT), and incubated at 32 or 22°C under low agitation. The cultures harvested at the mid-exponential phase of bacterial growth (OD_600_ = 0.2). Two aliquots (50 mL) of each biological replicate were harvested for RNA and protein extraction.

### Transcriptomic approach

#### RNA extraction

For RNA extraction, a culture volume of 50 mL for each biological triplicate was immediately centrifuged at 12,000 × *g* for 30 min at 4°C. Bacterial pellets were resuspended in 2 mL of RNAlater (Life Technologies, Carlsbad, USA), incubated at room temperature for 5 min, and then stored at −70°C overnight. The mixture was centrifuged at 12,000 × *g* for 10 min at 4°C and the pellets were lysed mechanically using a pestle. Total RNA was extracted using TRIzol RNA Isolation Reagent (Invitrogen, Carlsbad, USA) according to the manufacturer's instructions. The extracted RNA of each replicate was treated using the Turbo DNA-free kit (Ambion, Carlsbad, USA) and 1 μL was subjected to GBS-specific PCR (Mata et al., 2004) to confirm the absence of genomic DNA. The extracted RNA was quantified using a NanoDrop spectrophotometer (Thermo Scientific, Wilmington, USA) and its quality and integrity were evaluated using TapeStation 2200 (Agilent Technologies, Santa Clara, USA). RNA samples with RNA integrity number (RIN) values ranging from 7.7 to 9.1 were used.

#### RNA labeling and cRNA synthesis

In total, 50 ng of RNA from each biological replicate was amplified and Cy3-labeled using the Agilent Quick Amp Labeling kit (Agilent Technologies) along with RNA spike-in controls according to the Agilent One-Color Microarray-Based Expression Analysis protocol (Agilent Technologies). The resulting cRNA of each replicate was purified using an RNAeasy Mini Kit (Qiagen, Valencia, USA) according to the manufacturer's instructions. The cRNA concentration was measured using a NanoDrop spectrophotometer.

#### Hybridization and microarray analysis

The cRNA fragmentation and hybridization were performed using an Agilent Gene Expression Hybridization kit (Agilent Technologies) according to the manufacturer's instructions. Briefly, 600 ng of Cy3-labeled cRNA, with specific activity ≥16.2 pmol Cy3/ng was fragmented at 60°C for 30 min in a mix composed of 5 μL of 10X Blocking agent, 1 μL of 25X Fragmentation Buffer, and nuclease-free water to reach a final volume of 25 μL. After fragmentation, 25 μL of 2X GEx Hybridization Buffer was added to each replicate, and centrifuged at 21,900x *g* for 2 min. Next, 45 μL of hybridization solution was dispensed onto a custom-made Agilent slide (8 × 60K) formulated based on a library of 4,673 non-redundant genes (Agilent.SingleColor.72627) from nine fish GBS strains (SA07, SA20, SA53, SA288, SA289, SA320, 138P, GD201008_001, ZQ0910) for microarray-based gene expression analysis. Each microarray was created with two probes for each gene. The oligonucleotide microarray slide was designed using the eArray server (https://earray.chem.agilent.com/earray/) with the objective to perform comparative transcriptomic studies with different fish GBS strains. The slide was incubated at 65°C for 18 h at 10 rpm in a hybridization oven (Agilent Technologies). The slides were then washed in two buffers (Gene Expression Wash Buffer 1 and 2, both Agilent Technologies) and scanned using the Agilent DNA Microarray Scanner (Agilent Technologies). The data obtained from array images were extracted using Agilent Features Extraction software version 11.5 (Agilent Technologies).

Data analysis was performed with GeneSpring GX version 11.0.2 software (Agilent Technologies) using the workflow type “find differentially expressed genes.” The processed raw signal intensity for all probes was adjusted with a percentile shift normalization (percentile target = 75). Sample quality was assessed using a box plot to compare the intensity distributions of all replicates. A correlation matrix was used to compare reproducibility across the biological replicates. Principal component analysis (PCA) was used to assess the variability in replicates among temperatures from all genes detected. These quality assessments of transcriptomic data were performed in the workflow analysis, which is incorporated in GeneSpring. Normalized data were then filtered to retain probe sets with present (acceptable flag) signal intensity values in at least two of the three biological replicates in any one out of two conditions. Statistical analyses were performed on filtered data using an unpaired *t*-test. Genes with *p* < 0.05 were considered statistically significant. A fold-change cut-off of 2.0 was set to identify a differentially expressed gene with statistical significance between the two temperatures evaluated. Hierarchical clustering analysis was performed with genes showing *p* < 0.05 to arrange replicates into groups based on gene expression levels. All microarray data were deposited to the Gene Expression Omnibus Database under accession number GSE112416.

#### qPCR validation analysis

Four differentially expressed genes (DEGs) were selected for further validation by qPCR (Supplementary Table 1). The primers were designed using Primer Express 3.0 software (Life Technologies) and synthesized by Integrated DNA Technologies (IDT, Coralville, USA). The same three biological replicates of treated RNA that were used for microarray analysis for both temperatures were reverse transcribed into cDNA using SuperScript III reverse transcriptase kit (Invitrogen), according to the manufacturer's instructions. qPCR was performed using a GoTaq qPCR Master Mix (Promega) intercalating dye kit in a final volume of 20 μL containing 10 μL of 1 × Master Mix, 0.5 μM of primers (Supplementary Table 1), 0.2 μL of CXR reference dye, and 50 ng of cDNA. The qPCR assay was performed using a ViiA 7 Real-Time PCR System (Life Technologies) with the following cycle protocol: an initial step at 50°C for 2 min followed by 1 cycle of 95°C for 10 min, and 40 cycles of 95°C for 15 s and 60°C for 1 min. The relative mRNA expression of evaluated genes was normalized to the *S. agalactiae* reference genes *gyrA* and *recA* (Florindo et al., 2012; Faralla et al., 2014) using the ΔΔCt method (Livak and Schmittgen, 2001). Data acquisition and analysis were performed using ViiA 7 Software v.1.2.3 (Life Technologies). The Cohen/Kappa test was used to measure concordance between the microarray and qPCR results (Viera and Garrett, 2005).

### Proteomic approach

#### Protein extraction and tryptic digestion

For protein extraction, a culture volume of 50 mL for each biological triplicate was immediately centrifuged at 16,100x *g* for 20 min at 4°C and washed thrice with 50 mM Tris-HCl (pH 7.5). The pellets were then resuspended in 1 mL of lysis buffer [42% (w/v) urea, 15% thiourea, 4% sodium deoxycholate (SDC), 12.5 mM Tris-HCl pH 7.5, 1.5% dithiothreitol (DTT)] containing 1% protease inhibitor mix (GE Healthcare, Pittsburgh, USA). The samples were then incubated on ice for 15 min and sonicated on ice using a cell ultrasonic disruptor (Unique, Indaiatuba, Brazil), which was run for 1 min at maximum power (495 W) and stopped for 1 min in cycles until 20 min. The lysates were centrifuged at 21,900x *g* for 40 min at 4°C. The supernatant was collected, loaded into a Vivaspin 500 column with a threshold of 10 kDa (GE HealthCare), concentrated, and washed five times with 50 mM NH_4_HCO_3_. After washing, the concentrated samples were quantified on a Qubit 2.0 fluorometer (Invitrogen, Oregon, USA) using a Qubit protein assay kit (Molecular Probes, Oregon, USA).

For tryptic digestion, 50 μL (2 μg/μL) of each protein extract was mixed with 10 μL of 50 mM NH_4_HCO_3_, denatured with 25 μL of 0.2% RapiGest SF surfactant (Waters, Milford, USA), and incubated at 80°C for 15 min. After this, 2.5 μL of 100 mM DTT was added and heated at 60°C for 30 min. Afterwards, 2.5 μL of 300 mM iodoacetamine was added and the samples were kept at room temperature in a dark chamber for 30 min. The proteins were then enzymatically digested with 10 μL (0.5 μg/μL) trypsin (Promega, Madison, USA) and incubated at 37°C for 18 h. Following incubation, 10 μL of 5% trifluoroacetic acid (TFA; Sigma Aldrich, Saint Louis, USA) was added to each sample and incubated at 37°C for 90 min. The resulting peptide extracts were centrifuged at 21,900x *g* for 30 min at 6°C. Removal of SDC was performed by two-phase solvent extraction with ethyl acetate (Sigma Aldrich; 2:1) followed by addition of 0.5% TFA and centrifugation at 15,000x *g* for 5 min at 20°C. After centrifugation, the aqueous phase was collected and desalted using C18 MacroSpin Columns (Harvard Apparatus, Holliston, USA), according to the manufacturer's instructions. The samples were dried under vacuum in a Vacufuge Concentrator (Eppendorf, Hamburg, Germany), resuspended in 100 μL of 20 mM ammonium formate (Sigma Aldrich), transferred to Waters Total Recovery vials (Waters), and stored at −70°C until use.

#### NanoUPLC-HDMS^E^ Analysis

Biological replicates were analyzed by LC-MS using a nanoACQUITY ultra-performance liquid chromatography (UPLC) system coupled to a Synapt G2-Si HDMS mass spectrometer (Waters). Peptides were separated on an ACQUITY UPLC M-Class HSS T3 (1.8, 75 μm × 150 mm–pH 3) column used with a reversed-phase M-Class BEH C18 (5, 300 μm × 50 mm–pH 10) column (Waters). Analytical column temperature was maintained at 55°C. Bidimensional nanoUPLC tandem nano electrospray high definition mass spectrometry (nanoESI-HDMS^E^) using multiplexed data-independent acquisition (DIA) experiments were conducted using a reverse-phase gradient from 7 to 40% (v/v) acetonitrile (0.1% v/v formic acid) with a simulated 1D analysis and a delivery of 500 nL.min^−1^ in a nanoACQUITY UPLC 2D Technology system over 60 min (Gilar et al., 2005). Stoichiometric measurements based on scouting runs of the integrated total ion account (TIC) prior to analysis were performed to ensure standardized molar values across all samples. Typical on-column sample loads were 500 ng of total protein digests for each of the five fractions (500 ng per fraction/load).

For each measurement, the mass spectrometer was operated in resolution mode with a typical *m/z* resolving power of at least 25,000 full width at half maximum (FWHM), an ion mobility cell filled with helium gas, and a cross-section resolving power of at least 40 Ω/Δ Ω (Lalli et al., 2013). Analyses were performed using nano-electrospray ionization in the positive ion mode nanoESI (+) and a NanoLock-Spray ionization source (both from Waters). The lock mass channel was sampled every 30 s. The eluent was sprayed via PicoTip Emitters (Waters) at a spray voltage of 2.8 kv, sampling cone voltage of 30 v, and source offset of 30 v. The source temperature was set at 70°C. The time-of-flight analyzer of the mass spectrometry was calibrated with an MS/MS spectrum of GFP. Final instrument calibration was obtained by the double charged precursor ion [M + 2H]^2+^ = 785.8426 signal. The exact mass signals from multiplexed ion-mobility DIA scanning (HDMS^E^) were collected in an alternating low energy and elevated energy acquisition mode. Mass spectrometric analysis of tryptic peptides was performed using a mass spectrometer equipped with a T-Wave-IMS device in MS^E^ and HDMS^E^ modes as described previously (Distler et al., 2014). The radio frequency offset (MS profile) was adjusted such that the nanoESI-HDMS^E^ data were effectively acquired from *m/z* 400 to 2,000 by the MassLynx v.4.1 software (Waters), ensuring that any masses that were observed in the high energy spectra of < *m/z* 400 arose from dissociations in the collision cell.

#### Protein identification and quantitation

HDMS^E^ raw data of samples were processed using Progenesis QI for Proteomics (QIP) v.2.0 (Nonlinear Dynamics, Newcastle, UK), following previously described methods (Kuharev et al., 2015). Imported runs were used for automatic data processing for protein identification and quantitative information using dedicated algorithms in Progenesis QIP. The following parameters were used: peak picking limits = 5; maximum charge retention time limits = 8.

For peptide identification, data from three biological replicates of the strain in two conditions were searched against the *S. agalactiae* strain SA53 in-house database compiled from the annotated protein.fasta file. Using the database management tool of the ProteinLynx Global Server (PLGS) software v.3.0.2 (Waters), the sequence of each protein was reversed during the database queries and appended to the original database to assess the false positive rate during identification. The following parameters were used for peptide identification in Progenesis: digest reagent = trypsin; maximum missed cleavage = one; maximum protein mass = 600 kDa; modifications: carbamidomethyl of cysteine (fixed), acetyl N-terminal (variable), phosphoryl (variable), oxidation of methionine (variable); search tolerance parameters: peptide tolerance = 10 ppm, fragment tolerance = 20 ppm, maximum false discovery rate (FDR) = 4%; ion matching requirements used default parameters: fragments per peptide = 1, fragments per protein = 3, peptide per protein = 1.

Protein-level quantitation was performed by relative quantitation using an Hi-N algorithm incorporated in Progenesis. Peptides identified with a score ≤ 3, mass error ≥20 ppm, and sequence length ≤ 6 amino acids were removed. Proteins identified with at least two peptides (with ≥1 proteotypic peptide per protein) and present in at least two of three biological replicates for the GBS strain were considered. A protein was considered differentially expressed at 32°C in relation to 22°C if there was also a significant (*p* ≤ 0.05, ANOVA) change in expression with ≥ two-fold (log_2_ ratio ≥1.0). To obtain a general overview of protein expression among strains in different conditions, PCA and hierarchical clustering analyses were performed using *ggbiplot* version 0.55 (Vu, 2011) and *gplots* version 3.0.1 (Warnes et al., 2016) packages, respectively, in R software version 3.4.1 (R Core Team, 2013). PCA plot was performed with all the proteins detected in our data, whereas the heatmap plot only used proteins if there was significance (*p* ≤ 0.05, ANOVA). The MS proteomics data are available at the ProteomeXchange Consortium via the PRIDE (Vizcaíno et al., 2016) partner repository under the identifier PXD009330.

### Bioinformatic analyses

The transcripts and proteins identified for SA53 under both temperature conditions were analyzed using the prediction tools SurfG+ version 1.0.2 (Barinov et al., 2009) and Cluster of Orthologous Genes (COG) version 2014 db (Galperin et al., 2015) to predict subcellular localization and orthologous groups by functional category, respectively. COG database search was performed using an in-house script (available at: https://github.com/aquacen/blast_cog). Interactions among genes/proteins identified as differentially expressed at 32°C were analyzed using STRING web tool version 10.5 (Szklarczyk et al., 2017) with *Streptococcus agalactiae* NEM316 as reference and allowed experimental confidences and interaction score ≥0.700 (high confidence). The interaction networks obtained were visualized using Cytoscape version 3.5.1 (Shannon et al., 2003).

### Correlation analysis between transcriptomic and proteomic data

To determine the correlation between expressed genes and proteins from our results, the expression level of a transcript with statistical significance (*p* < 0.05) was correlated with the abundance of the corresponding protein (also *p* < 0.05) present in the proteomic dataset. Pearson's correlation was calculated using R software version 3.4.1 (R Core Team, 2013).

## Results

### Transcriptome analysis

The effect of temperature on the GBS transcriptome was evaluated using a whole-genome DNA microarray. We identified a total of 1,730 transcripts in SA53, with at least 98% identity with the probes of the array, characterizing 94.9% of the predicted genome of the strain (Supplementary Table 2). The same transcripts were identified at both temperatures, but differences in transcriptome intensity values were observed during bacterial growth at 22 and 32°C. The quality of transcriptomic data showed small variations among replicates at intensity values (Supplementary Figure 1A) with a correlation coefficient >95% (Supplementary Figure 1B), demonstrating a high reproducibility of transcriptome profiles. Additionally, in the PCA analysis, data from 22 and 32°C could be discriminated (Supplementary Figure 1C).

**Figure 1:**
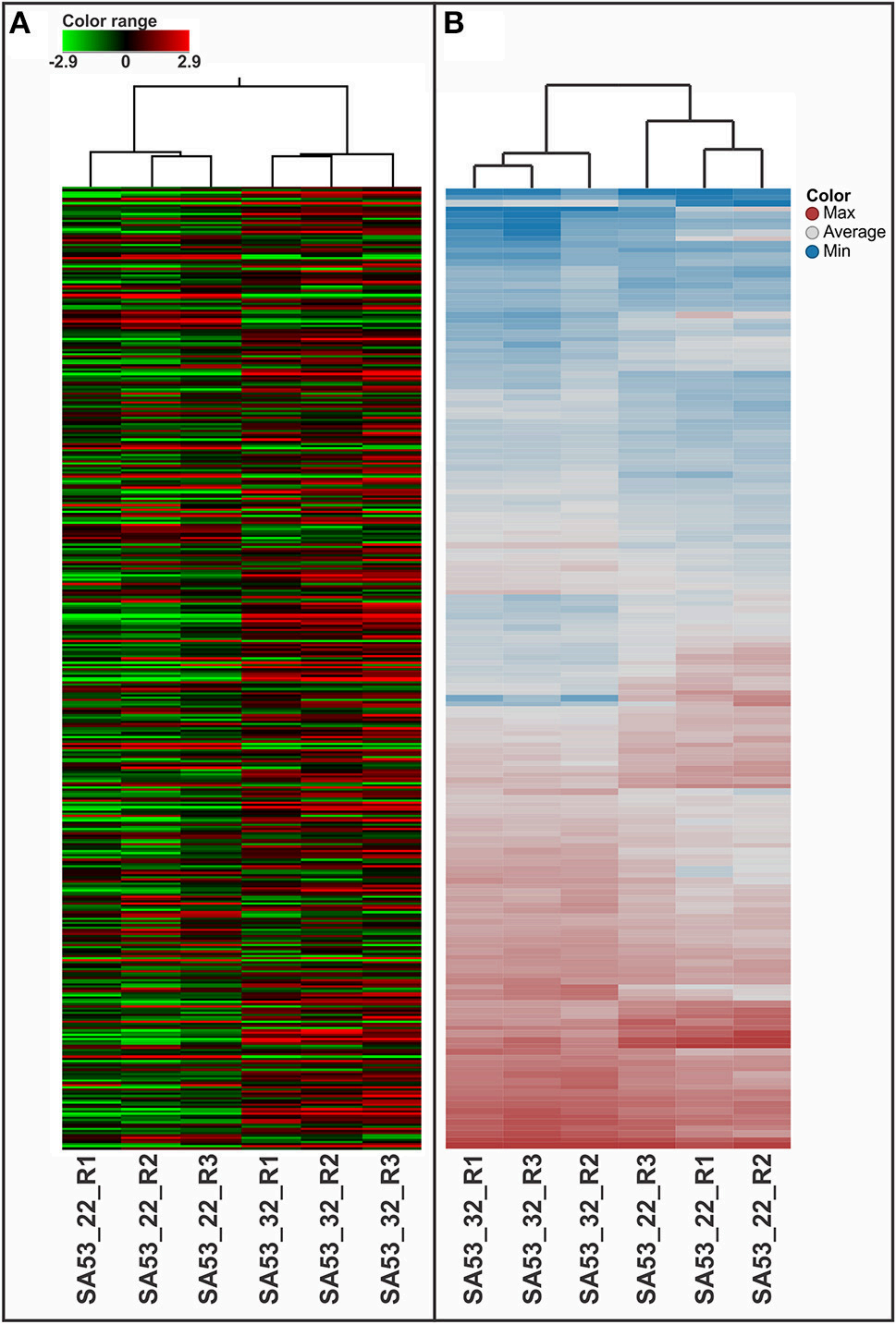
Heatmap analysis for biological triplicates of GBS strains tested at 22 and 32°C, as evaluated by microarray **(A)** and LC-HDMS^E^ **(B)**.

The 1,730 transcripts originally identified in two out of three replicates were reduced to 579 transcripts with *p* ≤ 0.05. Hierarchical clustering of these transcripts showed that the strain has a distinct transcriptomic pattern influenced by temperature, demonstrating a relationship in gene expression profiling across replicates with temperature (Figure 1A). Among these transcripts, 75 were upregulated at 32°C, whereas 32 were downregulated at 32°C (Table 1). These differentially expressed genes represented ~6% of the genome of the SA53 strain.

**Table 1:** Transcripts identified as differentially regulated at 32°C compared to 22°C.

**Accession**	**Gene**	**Fold change**	**Functional category^a^**	**Subcellular localization^b^**
SaSA53_0098	Aspartokinase	−2.53	E	CYT
SaSA53_0175	Phosphoribosylformylglycinamidine synthase	−2.28	F	CYT
SaSA53_0177	purM Phosphoribosylformylglycinamidine cyclo-ligase	−2.08	F	CYT
SaSA53_0180	purH Bifunctional purine biosynthesis protein	−2.02	F	CYT
SaSA53_0194	purD Phosphoribosylamine–glycine ligase	−2.15	F	CYT
SaSA53_0196	purK N5-carboxyaminoimidazole ribonucleotide synthase	−2.11	F	CYT
SaSA53_0583	Phosphate ABC transporter ATP-binding protein	−4.17	P	CYT
SaSA53_0584	Membrane protein	−4.20	P	MEM
SaSA53_0827	noxE NADH oxidase	−2.10	I	CYT
SaSA53_0850	Hypothetical protein	−2.02	K	CYT
SaSA53_0860	Ribosomal RNA small subunit methyltransferase B	−2.07	J	MEM
SaSA53_0930	apbE Thiamine biosynthesis protein	−2.09	H	CYT
SaSA53_0931	NADPH-dependent FMN reductase	−2.11	C	CYT
SaSA53_0932	NADPH-dependent FMN reductase	−2.12	S	CYT
SaSA53_0936	guaC GMP reductase	−2.00	F	CYT
SaSA53_1044	Branched-chain amino acid ABC transporter permease	−2.04	R	MEM
SaSA53_1244	Glutamine ABC transporter permease	−2.18	ET	PSE
SaSA53_1245	Peptide ABC transporter ATP-binding protein	−2.30	E	CYT
SaSA53_1300	Peptidylprolyl isomerase	−2.27	O	PSE
SaSA53_1432	Isochorismatase	−2.06	HR	CYT
SaSA53_1640	Adhesion protein	−2.25	P	PSE
SaSA53_1651	PTS mannose transporter subunit IID	−4.15	G	MEM
SaSA53_1652	PTS mannose transporter subunit IIC	−4.18	G	MEM
SaSA53_1653	PTS mannose transporter subunit IIB	−4.77	G	CYT
SaSA53_1654	PTS mannose transporter subunit IIA	−5.44	G	CYT
SaSA53_1706	cAMP factor	−2.04	R	SEC
SaSA53_1710	metF Methylenetetrahydrofolate reductase	−4.84	E	CYT
SaSA53_1711	metE 5-methyltetrahydropteroyltriglutamate– homocysteine methyltransferase	−4.04	E	CYT
SaSA53_1735	ABC transporter ATP-binding protein	−3.81	R	CYT
SaSA53_1736	ABC transporter permease	−3.74	R	MEM
SaSA53_1737	ABC transporter substrate-binding protein	−2.96	R	PSE
SaSA53_1745	nrdD Anaerobic ribonucleoside-triphosphate reductase	−2.16	F	CYT
SaSA53_0015	Hypothetical protein	2.05	Q	CYT
SaSA53_0090	PTS cellobiose transporter subunit IIA	5.62	G	CYT
SaSA53_0091	Cytochrome C biogenesis protein CcmE	5.88	G	SEC
SaSA53_0092	PTS system. cellobiose-specific IIC component	7.00	G	MEM
SaSA53_0095	Competence protein	2.02	L	CYT
SaSA53_0100	Enoyl-CoA hydratase	3.20	I	CYT
SaSA53_0104	2-nitropropane dioxygenase	2.07	R	CYT
SaSA53_0105	fabD Malonyl CoA-acyl carrier protein transacylase	2.08	I	CYT
SaSA53_0265	Single-stranded DNA-binding protein	2.10	L	CYT
SaSA53_0315	comX Competence-specific sigma factor	2.11	K	CYT
SaSA53_0319	hrcA Heat-inducible transcription repressor	2.13	K	CYT
SaSA53_0320	grpE Protein	2.03	O	CYT
SaSA53_0336	Pyridine nucleotide-disulfide oxidoreductase family protein	2.11	C	CYT
SaSA53_0344	Permease	2.12	V	MEM
SaSA53_0373	oppC Oligopeptide transport system permease protein	2.17	EP	MEM
SaSA53_0374	oppD Oligopeptide transport ATP-binding protein	2.17	EP	CYT
SaSA53_0375	oppF Oligopeptide transport ATP-binding protein	2.24	E	CYT
SaSA53_0387	Hypothetical protein	2.14	R	CYT
SaSA53_0472	MutT/nudix family protein	2.75	V	CYT
SaSA53_0477	Phosphoglucomutase	2.19	G	CYT
SaSA53_0507	Acetoin reductase	2.49	IQR	CYT
SaSA53_0551	Hypothetical protein	2.04	M	CYT
SaSA53_0581	DNA-entry nuclease	2.57	L	SEC
SaSA53_0589	Glucuronide permease	4.04	G	MEM
SaSA53_0590	2-dehydro-3-deoxygluconokinase	2.23	G	CYT
SaSA53_0593	uxaC Uronate isomerase	2.98	G	CYT
SaSA53_0594	uxuA Mannonate dehydratase	2.01	G	CYT
SaSA53_0660	MFS transporter	2.26	P	MEM
SaSA53_0691	nagB Glucosamine-6-phosphate deaminase	2.21	G	CYT
SaSA53_0801	msrB Peptide methionine sulfoxide reductase	2.07	O	CYT
SaSA53_0919	Hypothetical protein	2.09	P	MEM
SaSA53_0954	Hypothetical protein	5.49	I	CYT
SaSA53_0955	Hypothetical protein	5.92	G	MEM
SaSA53_0956	Hypothetical protein	7.56	G	MEM
SaSA53_0957	Hypothetical protein	7.95	R	CYT
SaSA53_0982	BCCT family transporter	2.07	M	MEM
SaSA53_1114	ABC transporter	2.09	V	CYT
SaSA53_1215	Ammonium transporter	2.24	P	MEM
SaSA53_1251	Cell wall surface anchor protein	2.04	–	PSE
SaSA53_1268	Transglutaminase	2.18	D	CYT
SaSA53_1269	Hypothetical protein	3.01	P	CYT
SaSA53_1281	Hypothetical protein	2.22	S	PSE
SaSA53_1287	Hypothetical protein	3.84	EP	CYT
SaSA53_1288	Peptide ABC transporter ATP-binding protein	3.49	EP	CYT
SaSA53_1289	Peptide ABC transporter permease	4.11	EP	MEM
SaSA53_1290	Peptide ABC transporter permease	4.59	EP	MEM
SaSA53_1291	Nickel ABC transporter substrate-binding protein	3.09	E	PSE
SaSA53_1373	Amidase	2.26	J	CYT
SaSA53_1406	Dihydroxyacetone kinase	2.21	K	CYT
SaSA53_1407	Dihydroxyacetone kinase subunit K	2.99	G	CYT
SaSA53_1408	Hypothetical protein	2.84	R	CYT
SaSA53_1409	PTS mannose transporter subunit IID	2.89	T	CYT
SaSA53_1410	Glycerol transporter	2.09	G	MEM
SaSA53_1412	Hypothetical protein	2.45	G	CYT
SaSA53_1431	3-hydroxybutyryl-CoA dehydrogenase	2.90	I	CYT
SaSA53_1435	Universal stress protein	2.16	T	CYT
SaSA53_1460	Multidrug MFS transporter	3.00	GEPR	MEM
SaSA53_1469	trx Thioredoxin	2.01	O	CYT
SaSA53_1518	Acid phosphatase	3.63	R	PSE
SaSA53_1549	Glycine/betaine ABC transporter permease	2.74	E	PSE
SaSA53_1550	Glycine/betaine ABC transporter ATP-binding protein	3.33	E	CYT
SaSA53_1561	tal Transaldolase	2.30	G	CYT
SaSA53_1564	ulaD 3-keto-L-gulonate-6-phosphate decarboxylase	2.93	G	CYT
SaSA53_1565	PTS ascorbate transporter subunit IIA	4.00	GT	CYT
SaSA53_1566	PTS ascorbate transporter subunit IIB	4.49	G	CYT
SaSA53_1567	PTS ascorbate transporter subunit IIC	3.76	G	MEM
SaSA53_1598	PTS system N-acetylgalactosamine-specific transporter subunit IIC	2.45	G	MEM
SaSA53_1599	PTS system N-acetylgalactosamine-specific transporter subunit IIB	2.12	G	CYT
SaSA53_1600	Glucuronyl hydrolase	2.47	G	CYT
SaSA53_1601	PTS system N-acetylgalactosamine-specific transporter subunit IIA	2.36	G	CYT
SaSA53_1602	Gluconate 5-dehydrogenase	2.93	IQR	CYT
SaSA53_1603	Hypothetical protein	3.79	G	CYT
SaSA53_1604	2-keto-3-deoxygluconate kinase	3.87	G	CYT
SaSA53_1605	2-dehydro-3-deoxyphosphogluconate aldolase	2.45	G	CYT
SaSA53_1791	sdhA L-serine dehydratase. iron-sulfur-dependent. alpha subunit	2.02	E	CYT

a *Orthologous groups by functional category: C, Energy production and conversion; D, Cell cycle control, cell division, chromosome partitioning; E, Amino acid transport and metabolism; F, Nucleotide transport and metabolism; G, Carbohydrate transport and metabolism; H, Coenzyme transport and metabolism; I, Lipid transport and metabolism; J, Translation, ribosomal structure and biogenesis; K, Transcription; L, Replication, recombination and repair; M, Cell wall/membrane/envelope biogenesis; O, Post-translational modification, protein turnover, and chaperones; P, Inorganic ion transport and metabolism; Q, Secondary metabolites biosynthesis, transport, and catabolism; R, General function prediction only; S, Function unknown; T, Signal transduction mechanisms; V, Defense mechanisms; -, not determined*.

b *Subcellular localization: CYT, cytoplasmic; PSE, potentially surface-exposed; MEM, membrane; SEC, secreted*.

**Figure 2:**
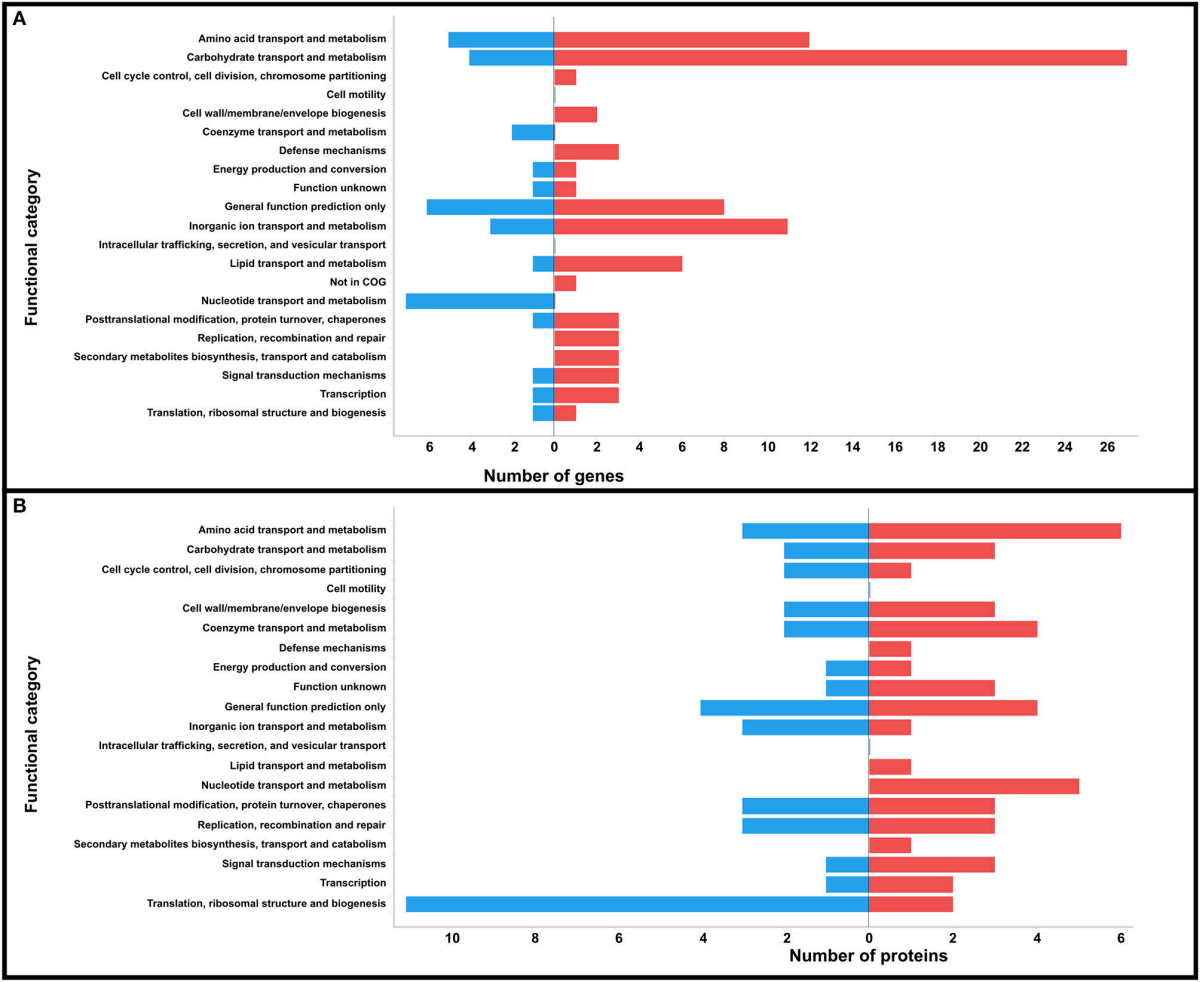
Prediction of COG functional categories of the DEGs **(A)** and DPAs **(B)** identified in strain SA53 at the two temperatures tested. Blue bar represents downregulation and red bar represents upregulation at 32°C.

In accordance with the subcellular localization analysis, these DEGs were classified as cytoplasmic (*n* = 73), membrane (*n* = 22), potentially surface-exposed (PSE) (*n* = 9), and secreted (*n* = 3), with ~34 and ~30% being bacterial surface-related when downregulated and upregulated at 32°C, respectively (Table 1). The DEGs were classified using COG on 18 functional categories (Table 1 and Figure 2A). A higher number of genes related to metabolism were upregulated at 32°C, except those involved in nucleotide metabolism (F) and coenzyme transport and metabolism (H), which were exclusively downregulated at 32°C. On the other hand, genes involved in cell cycle control (D), cell wall biogenesis (M), defense mechanisms (V), replication (L), and secondary metabolites biosynthesis (Q) were exclusively upregulated at 32°C.

From 38 genes considered to be associated with virulence (Glaser et al., 2002; Mereghetti et al., 2008) in the SA53 genome, 37 were detected by transcriptomic analysis (Table 2). Among them, 13 showed statistical significance, including cAMP factor, *lmb*, and *noxE*, which were downregulated at 32°C. The other putative virulence genes (cell wall surface anchor protein, *bibA, fbsA, hylB, cpsB, cpsC, cpsD, rmlA, pbp2A*, and *pbpX*) did not vary in expression between the evaluated temperatures (Table 2). In our transcriptomic dataset, we identified stress protein genes (*hrcA, grpE*, universal stress protein, and *trx*) that were upregulated at 32°C.

**Table 2:** Known GBS virulence factors and their identification and regulation at 32°C compared to 22°C in transcriptomic and proteomic analysis.

**Virulence factor**	**Accession**	**Transcript detected^a^**	**Regulation^c^**	**Protein detected^b^**	**Regulation^c^**
**ADHESION**					
Elongation factor Tu	SaSA53_0653	Yes	NS	Yes	NS
Cell wall surface anchor protein	SaSA53_0662	Yes	Unchanged	Yes	NS
GapC	SaSA53_1519	Yes	NS	Yes	NS
GapN	SaSA53_0716	Yes	NS	Yes	Unchanged
PavA	SaSA53_1046	Yes	NS	Yes	NS
BibA	SaSA53_1722	Yes	Unchanged	No	
FbsA	SaSA53_0903	Yes	Unchanged	No	
Lmb	SaSA53_1640	Yes	Down	Yes	NS
Pi-2b	SaSA53_1187	Yes	NS	No	
**INVASION**					
CylE	SaSA53_0568	No		No	
cAMP factor	SaSA53_1706	Yes	Down	Yes	Up
Hemolysin A	SaSA53_0483	Yes	NS	Yes	NS
Eno	SaSA53_0533	Yes	NS	Yes	NS
IagA	SaSA53_0601	Yes	NS	Yes	Up
Internalin	SaSA53_0799	Yes	NS	No	
HylB	SaSA53_1053	Yes	Unchanged	Yes	NS
NoxE	SaSA53_0827	Yes	Down	Yes	NS
**IMMUNE EVASION**					
Capsular polysaccharide–CpsG	SaSA53_1029	Yes	NS	Yes	NS
Capsular polysaccharide–CpsF	SaSA53_1030	Yes	NS	Yes	NS
Capsular polysaccharide–CpsE	SaSA53_1031	Yes	NS	Yes	NS
Capsular polysaccharide–CpsD	SaSA53_1032	Yes	NS	Yes	NS
Capsular polysaccharide–CpsC	SaSA53_1033	Yes	Unchanged	Yes	NS
Capsular polysaccharide–CpsB	SaSA53_1034	Yes	Unchanged	Yes	Up
SodA	SaSA53_0678	Yes	Unchanged	Yes	NS
ScpB	SaSA53_0381	Yes	NS	Yes	Down
Group B antigen–RmlB	SaSA53_1054	Yes	NS	Yes	NS
Group B antigen–RmlC	SaSA53_1055	Yes	NS	Yes	NS
Group B antigen–RmlA	SaSA53_1056	Yes	Unchanged	Yes	NS
Sip	SaSA53_0182	Yes	NS	Yes	NS
Serine protease	SaSA53_1720	Yes	NS	Yes	NS
**MULTIDRUG RESISTANCE**					
DltD	SaSA53_1540	Yes	NS	Yes	NS
DltB	SaSA53_1542	Yes	NS	Yes	NS
DltA	SaSA53_1543	Yes	NS	Yes	NS
Pbp2A	SaSA53_1724	Yes	Unchanged	Yes	NS
PbpX	SaSA53_0050	Yes	Unchanged	Yes	NS
Pbp1A	SaSA53_0061	Yes	NS	Yes	NS
Pbp2B	SaSA53_0656	Yes	NS	Yes	NS
Beta-lactamase	SaSA53_0562	Yes	NS	Yes	NS

a *Transcripts identified in microarray analysis*.

b *Proteins identified in LC-HDMS^E^ analysis*.

c *Transcript or protein with p > 0.05 were considered as detected but not significant–NS; Transcript or protein with p < 0.05 were classified according to the fold-change as downregulated (Down), upregulated (Up), or unchanged*.

To better understand the biological functions of transcripts identified as differentially expressed at 32°C, an interactome analysis was conducted, revealing 97 interactions (Supplementary Figure 2A). The greatest number of interactions were verified in proteins related to the phosphoenolpyruvate-dependent phosphotransferase system (PTS; cluster 1), ABC transport system (cluster 2), ascorbate and aldarate metabolism (cluster 3), purine metabolism (cluster 4), and metabolic pathways (cluster 5) that were both downregulated and upregulated at 32°C (Supplementary Figure 2A).

In our validation analysis, we observed a perfect agreement (kappa coefficient = 1) between microarray and qPCR results in the direction of regulation (downregulation and upregulation) for all genes evaluated (Supplementary Table 1). Therefore, this result demonstrated that the microarray data are valid.

### Proteome analysis

The effect of temperature on the GBS proteome was evaluated using an LC-HDMS^E^ approach. In total, 29,790 peptides with a normal distribution of 10 ppm error were identified (Supplementary Figure 3A). Peptides as source fragments, with a charge state of at least [M +2H]^2+^, and absence of decoys were considered to increase data quality. Therefore, our proteomics analysis allowed the identification and quantitation of 1,046 proteins for SA53 (Supplementary Table 3), with an average of 28 peptides per protein and a calculated FDR = 0% when decoy detection was set at an agreement with two of the three replicates. These data characterized ~62% of the predicted proteome of SA53. Predicted proteins smaller than 10 kDa were also identified in the proteome, despite the threshold of the column used in protein extraction (see Material and Methods). In total, 1,043 proteins were present in both temperatures tested. In relation to protein content, more than 99% of the identified proteins were found in all three replicates of each temperature, showing a high reproducibility among the biological replicates (Supplementary Figure 3B). In the PCA analysis there was no clear variability in protein abundance among the evaluated temperatures (Supplementary Figure 3C). The dynamic range of the identified proteins reached ~4.5 log orders of magnitude between the most and least abundant proteins in each temperature tested. Supplementary Table 4 summarizes the ten most and least abundant proteins.

Label-free quantification was applied to evaluate the relative abundance of the proteome of the strain at low and high temperature conditions. In summary, 163 proteins had *p* ≤ 0.05, and 81 (4.7% of the predicted proteome) of them showed a difference in level of abundance in SA53, with 37 and 44 proteins downregulated and upregulated at 32°C compared to 22°C, respectively (Table 3). Beyond that, two proteins were exclusively identified only at 32°C, and one protein was exclusively identified only at 22°C (Table 3). The hierarchical clustering of significant proteins (*n* = 163) demonstrated the arrangement of biological replicates by temperature condition (Figure 1B), indicating that the strain had a distinct proteomic pattern influenced by temperature.

**Table 3:** Proteins identified as differentially regulated at 32°C compared to 22°C.

**Accession**	**Peptides**	**Score**	**Product**	**Fold change**	**Functional category^a^**	**Subcellular localization^b^**
SaSA53_0162	7	39.04	rnpA Ribonuclease P protein component	−4.34	J	CYT
SaSA53_0262	4	29.12	Thioredoxin	−2.78	O	CYT
SaSA53_0373	5	29.93	oppC Oligopeptide transport system permease protein	−4.08	EP	MEM
SaSA53_0381	20	111.21	Reticulocyte binding protein	−20.82	D	PSE
SaSA53_0468	4	20.23	RNA-binding protein	−9.57	R	CYT
SaSA53_0494	11	62.19	UDP-N-acetylmuramoylpentapeptide-lysine N(6)-alanyltransferase MurM	−2.73	M	CYT
SaSA53_0504	27	167.67	ftsX Cell division protein	−2.23	D	PSE
SaSA53_0506	7	44.70	Hypothetical protein	−2.94	R	CYT
SaSA53_0535	20	107.34	aroA 3-phosphoshikimate 1-carboxyvinyltransferase	−2.56	E	CYT
SaSA53_0591	22	161.80	GntR family transcriptional regulator	−3.38	K	CYT
SaSA53_0621	31	227.07	N5.N10-methylenetetrahydromethanopterin reductase	−2.08	HR	CYT
SaSA53_0627	14	95.62	lgt Prolipoprotein diacylglyceryl transferase	−3.53	M	MEM
SaSA53_0667	67	570.69	RNA helicase	−2.08	L	CYT
SaSA53_0689	25	183.91	queA S-adenosylmethionine:tRNA ribosyltransferase-isomerase	−2.49	J	CYT
SaSA53_0758	60	443.06	atpG ATP synthase gamma chain	−2.11	C	CYT
SaSA53_0767	94	702.74	pheT Phenylalanyl–tRNA ligase beta subunit	−3.45	J	CYT
SaSA53_0837	11	57.66	GNAT family acetyltransferase	−3.05	J	CYT
SaSA53_0855	45	342.98	Phosphate import ATP-binding protein PstB	−3.03	P	CYT
SaSA53_0857	6	30.89	Phosphate ABC transporter. permease protein PstA	−2.08	P	PSE
SaSA53_0910	5	28.06	Appr-1-p processing protein	−24.25	J	CYT
SaSA53_1178	23	151.58	Hypothetical protein	−2.77	S	CYT
SaSA53_1182	5	26.28	SAM-dependent methyltransferase	−74.54	J	CYT
SaSA53_1276	14	92.26	ybeY Endoribonuclease	−4.19	J	CYT
SaSA53_1291	8	39.72	Nickel ABC transporter substrate-binding protein	−3.22	E	PSE
SaSA53_1348	10	66.78	tRNA [cytidine(34)-2′-O]-methyltransferase	−2.31	J	CYT
SaSA53_1381	30	208.69	Primosomal protein DnaI	−2.53	L	CYT
SaSA53_1392	9	53.24	Cobalt ABC transporter permease	−13.08	H	MEM
SaSA53_1476	25	131.84	ATP-dependent DNA helicase RecD-like protein	−4.22	L	CYT
SaSA53_1484	6	46.76	Glycerol uptake permease	−14.72	G	MEM
SaSA53_1487	6	33.13	Crp/Fnr family transcriptional regulator	−4.53	T	CYT
SaSA53_1504	22	140.11	Hypothetical protein	−2.42	R	CYT
SaSA53_1522	30	242.33	rpsG 30S ribosomal protein S7	−2.80	J	CYT
SaSA53_1580	113	845.26	ATP-dependent Clp protease ATP-binding protein	−2.80	O	CYT
SaSA53_1624	10	54.84	dexB Glucan 1.6-alpha-glucosidase	−7.97	G	CYT
SaSA53_1675	30	187.15	Ribosomal RNA small subunit methyltransferase E	−2.41	J	CYT
SaSA53_1679	7	47.08	GNAT family acetyltransferase	−3.63	J	CYT
SaSA53_1734	18	190.32	groS 10 kDa chaperonin	−2.25	O	CYT
SaSA53_0041	77	778.26	tkt Transketolase	2.67	G	CYT
SaSA53_0177	14	90.81	purM Phosphoribosylformylglycinamidine cyclo-ligase	3.68	F	CYT
SaSA53_0180	44	342.70	purH Bifunctional purine biosynthesis protein	2.51	F	CYT
SaSA53_0196	16	108.09	purK N5-carboxyaminoimidazole ribonucleotide synthase	2.34	F	CYT
SaSA53_0197	22	136.77	Hypothetical protein	3.07	O	CYT
SaSA53_0268	5	24.57	lytR Sensory transduction protein	13.26	KT	CYT
SaSA53_0358	39	322.36	Amino acid ABC transporter ATP-binding protein	2.34	E	CYT
SaSA53_0466	17	142.16	sepF Cell division protein	2.90	D	CYT
SaSA53_0478	28	186.28	folD Bifunctional protein	2.20	H	CYT
SaSA53_0481	12	118.73	xseB Exodeoxyribonuclease 7 small subunit	3.22	L	CYT
SaSA53_0522	5	74.86	Flavodoxin	3.45	C	CYT
SaSA53_0601	15	92.29	Family 1 glycosyl transferase	4.11	M	CYT
SaSA53_0730	5	44.15	Hypothetical protein	3.36	M	SEC
SaSA53_0736	36	274.28	Phosphomethylpyrimidine kinase	3.31	H	CYT
SaSA53_0740	5	32.94	GNAT family acetyltransferase	2.07	JO	CYT
SaSA53_0811	106	1000.67	Pyruvate kinase	2.47	G	CYT
SaSA53_0815	5	26.89	Amino acid ABC transporter permease	3.05	E	MEM
SaSA53_0867	14	71.90	Hypothetical protein	2.98	S	CYT
SaSA53_0890	7	90.07	ESAT-6-like protein	2.00	S	CYT
SaSA53_0896	6	43.51	pyrC Dihydroorotase	2.88	F	CYT
SaSA53_0932	10	63.85	NADPH-dependent FMN reductase	3.55	S	CYT
SaSA53_0975	19	117.47	folP Dihydropteroate synthase	2.14	H	CYT
SaSA53_1034	32	245.18	cpsB Tyrosine-protein phosphatase	2.09	T	CYT
SaSA53_1091	3	23.67	Amino acid transporter	3.70	E	MEM
SaSA53_1101	39	260.34	3-hydroxy-3-methylglutaryl coenzyme A reductase	2.17	I	CYT
SaSA53_1156	22	219.87	Hypothetical protein	2.05	T	CYT
SaSA53_1239	7	33.05	Beta-1,6-galactofuranosyltransferase	2.54	M	CYT
SaSA53_1245	47	463.68	Peptide ABC transporter ATP-binding protein	2.02	E	CYT
SaSA53_1277	6	28.64	Tetracenomycin polyketide synthesis O-methyltransferase TcmP	2.04	Q	CYT
SaSA53_1325	2	11.05	PTS lactose transporter subunit IIC	2.28	G	MEM
SaSA53_1346	30	281.46	upp Uracil phosphoribosyltransferase	2.31	F	CYT
SaSA53_1383	9	84.65	nrdR Transcriptional repressor	2.07	K	CYT
SaSA53_1416	6	37.90	SAM-dependent methyltransferase	2.56	H	CYT
SaSA53_1440	27	181.27	recG ATP-dependent DNA helicase	2.96	L	CYT
SaSA53_1469	23	221.64	trx Thioredoxin	6.23	O	CYT
SaSA53_1502	10	77.30	Hypothetical protein	2.86	L	CYT
SaSA53_1585	19	157.05	Alkyl hydroperoxide reductase subunit C	2.07	V	CYT
SaSA53_1641	11	65.99	dtd D-aminoacyl-tRNA deacylase	4.00	J	CYT
SaSA53_1682	7	52.91	Hypothetical protein	2.42	E	CYT
SaSA53_1706	7	40.62	cAMP factor	2.75	R	SEC
SaSA53_1735	48	374.57	ABC transporter ATP-binding protein	3.53	R	CYT
SaSA53_1737	54	484.23	ABC transporter substrate-binding protein	4.53	R	PSE
SaSA53_1796	9	46.73	Energy-coupling factor transporter ATP-binding protein EcfA1	2.39	PR	CYT
SaSA53_1812	34	354.77	arcC Carbamate kinase	2.39	E	CYT
SaSA53_0353	2	17.02	Hypothetical protein		S	CYT
SaSA53_1139	5	32.18	Macrolide ABC transporter ATP-binding protein		M	CYT
SaSA53_1378	6	35.03	Hypothetical protein		R	CYT

a *Orthologous groups by functional category: C, Energy production and conversion; D, Cell cycle control, cell division, chromosome partitioning; E, Amino acid transport and metabolism; F, Nucleotide transport and metabolism; G, Carbohydrate transport and metabolism; H, Coenzyme transport and metabolism; I, Lipid transport and metabolism; J, Translation, ribosomal structure and biogenesis; K, Transcription; L, Replication, recombination and repair; M, Cell wall/membrane/envelope biogenesis; O, Post-translational modification, protein turnover, and chaperones; P, Inorganic ion transport and metabolism; Q, Secondary metabolites biosynthesis, transport, and catabolism; R, General function prediction only; S, Function unknown; T, Signal transduction mechanisms; V, Defense mechanisms*.

b *Subcellular localization: CYT, cytoplasmic; PSE, potentially surface-exposed; MEM, membrane; SEC, secreted*.

In total, 67, 7, 5, and 2 proteins with differential protein abundances (DPAs) were predicted as cytoplasmic, membrane, PSE, and secreted, respectively; of these, ~21 and ~13% were bacterial surface proteins downregulated and upregulated at 32°C, respectively (Table 3). According to COG analysis, the DPAs were classified into 18 categories (Table 3 and Figure 2B). Proteins involved in translation, ribosomal structure, and biogenesis (J), and inorganic ion metabolism (P) were the most abundant proteins that were downregulated at 32°C in relation to upregulated proteins. On the other hand, proteins involved in defense mechanisms (V), lipid metabolism (I), nucleotide metabolism, and secondary metabolites biosynthesis (Q) were exclusively identified as upregulated at 32°C.

We identified 33 known SA53 virulence factors by proteomic analysis (Table 2). Reticulocyte binding protein was downregulated at 32°C; IagA (Family 1 glycosyl transferase), CpsB, and cAMP factor were upregulated; and GapN did not vary in abundance between temperatures (Table 2). The other 28 putative virulence proteins, although detected, did not show differential abundance in our study. In addition, thioredoxin, which is a protein involved in oxidative stress, was also identified as downregulated at 32°C.

In order to study the main interactions among proteins identified as more abundant at 32°C compared to 22°C, we performed an interactome analysis, which revealed 26 protein-protein interactions. The greatest number of interactions was observed as upregulated at 32°C: PurH (SaSA53_0180) and PurM (SaSA53_0177), involved in purine metabolism; alkyl hydroperoxide reductase subunit C (SaSA53_1585) and thioredoxin (SaSA53_1469), involved in oxidative stress; pyruvate kinase (SaSA53_0811), related to glycolysis; and amino acid ABC transporter permease (SaSA53_0815), involved in amino acid metabolism (Supplementary Figure 2B).

### Correlation analysis

The Pearson correlation coefficient between gene expression levels and protein abundance for SA53 was 0.3390. This result showed that there was a low correlation between the transcriptome and proteome datasets in regard to the genes and proteins that were downregulated or upregulated in the same direction, for a given condition (low or high temperature).

## Discussion

Unlike human-adapted GBS, fish-adapted GBS strains are exposed to constant and abrupt changes in temperature during adaptation to aquatic environments and during the infection process as fish are poikilothermic animals and water in the environment can undergo rapid temperature changes due to short-term weather events. To better understand the characteristics of metabolism, adaptation, and pathogenicity of this agent in response to temperature variation, we analyzed the transcriptome and the proteome of a fish-adapted GBS strain subjected to two growth temperatures using microarray and LC-HDMS^E^ approaches.

The isolate selected for these analyses was the GBS SA53 strain. This strain was previously isolated from diseased Nile tilapia in Brazil (Mian et al., 2009) and belongs to ST-260, one of the most frequently identified genotypes in Latin America (Evans et al., 2008; Delannoy et al., 2013; Godoy et al., 2013; Barato et al., 2015; Barony et al., 2017). SA53 belongs to a group of fish-adapted genotypes that descend from a single branch unlike human GBS strains, and are going through reductive genome evolution (Barony et al., 2017). Its LD_50_ was not previously determined but isolates obtained from diseased fish have been demonstrated as highly virulent in experimental assays with Nile tilapia, with an LD_50_ ranging from 10^1^ to 10^5^ cfu mL^−1^ (Mian et al., 2009; Evans et al., 2015). Therefore, due the characteristics described above, SA53 was chosen to elucidate the gene expression and protein abundance in fish-adapted GBS strains at low (22°C) and high (32°C) temperature conditions, corresponding to temperatures showing low and high mortality rates in fish, respectively (Salvador et al., 2005; Mian et al., 2009; Amal et al., 2015; Al-Harbi, 2016; Mainardi et al., 2016; Chideroli et al., 2017).

For the temperatures analyzed in this study, the effect on transcriptome and proteome modification was not extensive, corroborating a previous study that compared the transcriptional response to temperature in one fish-adapted *Lactococcus garvieae* strain (Aguado-Urda et al., 2013). This suggests that fish-adapted GBS strains may be physiologically stable in the global gene expression and protein synthesis, even with rapid variation in environmental temperature, thus supporting the idea of possible adaptation of bacteria to the aquatic environment and the fish host. However, the expression of some important proteins was strongly modulated by the temperature shift, providing insights about the interaction of GBS with the fish host.

### Transcriptional response is modulated by temperature

**Figure 3:**
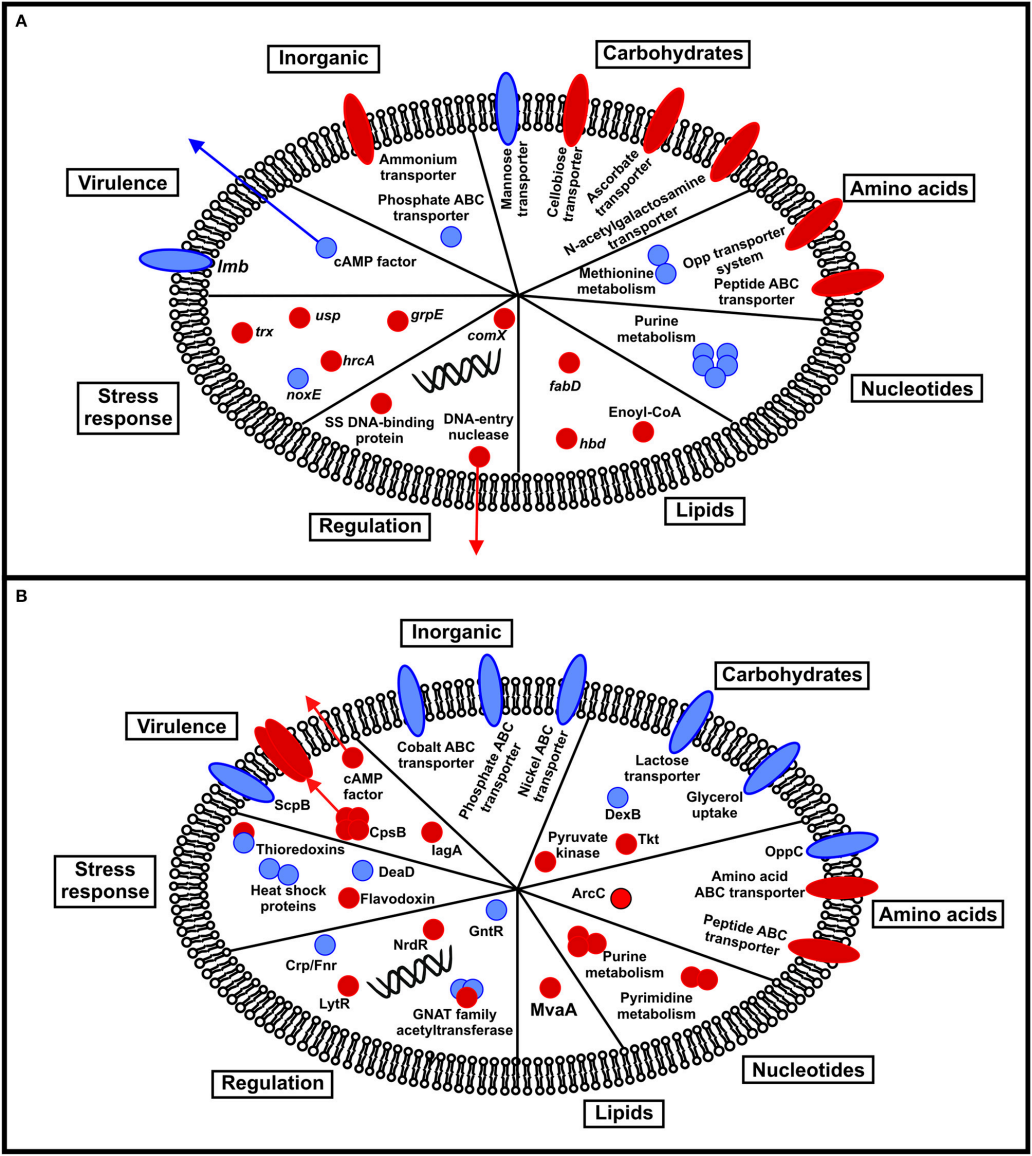
Schematic summarizing the transcriptional **(A)** and proteomic **(B)** response of fish-adapted GBS to incubation at different temperatures. Red circles indicate upregulation at 32°C, whereas blue circles represent downregulation at 32°C.

In our transcriptomic analysis, we observed that temperature could influence the expression of genes involved mainly in cellular metabolism (Figure 3A). Among genes that were downregulated at 32°C, we detected genes involved in purine metabolism: *purD, purH, purK, purM*, and *guaC*. These genes grouped together in our interactome analysis and are related to the purine biosynthetic pathway, described by Mereghetti et al. (2008) as responsible for the synthesis of inosine monophosphate, a compound important to satisfy the purine auxotrophic requirements of GBS (Rajagopal et al., 2005). A previous study demonstrated that purine metabolism in GBS is influenced by incubation temperature, being upregulated at 40°C compared to 30°C (Mereghetti et al., 2008). In our study, these transcripts were identified at 22°C and may be associated with an adaptive condition where the bacterium presumably increases its requirements of adenine and guanine during growth at low temperatures in an aquatic environment. The increase in uptake is achieved by either bacterial hydrolytic degradation of nucleic acids and nucleotides, or uptake of these nucleotides from the environment through utilization of the purine salvage pathway (Rajagopal et al., 2005). Utilization of this pathway is corroborated by detection of salvage enzymes *apt* (adenine phosphoribosyltransferase), *xpt* (xanthine phosphoribosyltransferase), hypoxanthine phosphoribosyltransferase, inosise-uride nucleoside N-ribohydrolase, and *add* (adenosine deaminase) in the transcriptome dataset.

Four genes involved in PTS mannose transport (subunit IIA, IIB, IIC, and IID) related to carbohydrate metabolism were downregulated at 32°C. This PTS system is considered important to the transport of mannose, fructose, and glucose for the species of genus *Streptococcus* (Pelletier et al., 1998; Abranches et al., 2003; Bidossi et al., 2012), through a mechanism that couples translocation (subunits IIC and IID) with phosphorylation (subunits IIA and IIB) of the carbon sources (Gutknecht et al., 1999). Mutation in subunits IIAB of *Streptococcus mutans* led to lower uptake of glucose and mannose in medium containing glucose as the sole carbohydrate source, whereas fructose uptake was not affected (Abranches et al., 2003). Fish-adapted GBS strains need to adjust their metabolism in response to nutrient availability in aquatic and host environments, especially in relation to glucose availability. At high temperature, a greater affinity for glucose is required for the growth of psychrotolerant and mesophilic bacteria (Nedwell, 1999). Exposure of GBS strains to glucose permits modulation of genes involved in cell envelope biogenesis and metabolism and transport of amino acids, ions, and other carbohydrates. However, high glucose availability can lead to decreased expression of genes involved in the uptake of carbohydrates and putative virulence factors (Di Palo et al., 2013). Thus, one can argue that fish-adapted GBS has a lower affinity for glucose at 22°C, increasing the expression of the PTS system, involved in mannose transport, which in turn promotes an increased capacity to import glucose for the bacterial cell. Nevertheless, future research needs to be conducted to evaluate the PTS mannose activity in fish-adapted GBS strains cultured in media containing different carbohydrate sources, and at different temperatures.

Genes involved in methionine metabolism, such as *metE* and *metF*, were also downregulated at 32°C. These genes are involved in the methionine synthesis pathway through methylation of homocysteine by *metE* in conjunction with *metF*, which then receives a methyl group from *folD* (methylenetetrahydrofolate dehydrogenase), to form methionine (Afzal et al., 2016). Genes involved in methionine synthesis have been considered critical for virulence in GBS (Shelver et al., 2003). However, in *Brucella melintesis*, mutation in one gene related to methionine synthesis culminated in reduced bacterial colonization in the spleen of a murine model (Lestrate et al., 2000). Together, these findings demonstrate that genes involved in methionine metabolism allow bacterial survival during infection. Methionine availability in fish blood, as well as fish body temperature, might influence the growth rate of GBS, predisposing the expression of virulence factors, and allowing the bacterium to resist clearance by the immune system, as previously verified by *in vitro* and *in vivo* assays with a human GBS strain (Shelver et al., 2003). However, further studies are needed to determine if *metE* and *metF* contribute to GBS virulence in fish and if they are essential for the growth of this bacterium in fish blood.

Metabolic pathways involved in the uptake of cellobiose, ascorbate, N-acetylgalactosamine, and amino acids were upregulated at 32°C. GBS strains also use cellobiose, ascorbate, and N-acetylgalactosamine as a carbon source (Glaser et al., 2002). Fish-adapted GBS strains do not contain cellulase genes in their genome and cellobiose uptake may occur during the infectious process in the gastrointestinal epithelium of fish, as the gastrointestinal tract is one of the major routes of GBS entry in fish (Iregui et al., 2016), or during adaptation to aquatic environments where cellobiose excreted via feces can be used as an alternative energy source. The genes involved in this type of metabolism (PTS cellobiose transporter subunit IIA and IIC) show a change in expression level of 5- to 7-fold when cultured at high temperature, thus interacting with each other in our interactome analysis.

Ascorbate can also be used as an alternative carbon source for many bacterial species including *E. coli, Klebsiella pneumoniae*, and *Streptococcus pneumoniae*, and its entry into bacterial cells occurs through the ascorbate-specific PTS system (Afzal et al., 2015). In our transcriptomic analysis, we identified four genes involved in ascorbic acid uptake (*ulaD*, PTS ascorbate transporter subunit IIA, IIB, and IIC) that interact with each other. Therefore, during the infectious process, the ascorbate contained in fish tissues may be used by GBS strains as an energy source for their growth and dissemination in this host, especially at high temperature.

N-acetylglucosamine is an important polysaccharide component in the cell wall of GBS strains (Pereira et al., 2013), and its catabolism provides organisms with a carbon and nitrogen source (Moye et al., 2014). N-acetylglucosamine was described as important for GBS adhesion to fish cells (Barato et al., 2016). Thus, expression of these genes at high temperature may contribute to adhesion and immune system evasion by fish-adapted GBS strains in aquatic hosts.

Additionally, among the genes upregulated at 32°C, six genes were involved in the transport of peptides including the Opp transport system (*oppCDF*), an important superfamily of conserved ATP-binding cassette transporters involved in bacterial nutrition, signaling, and virulence though internalization of peptides from the extracellular environment (Silva et al., 2017). In GBS, the Opp transport system is responsible for the uptake of oligopeptides in a nutritionally rich environment and contributes to infection by stimulating the adherence of pathogens to human cells and modulating fibrinogen-binding adhesin expression (Samen et al., 2004). However, there are no studies that demonstrate the participation of Opp genes in the pathogenesis of fish-adapted GBS strains.

Variations in temperature during bacterial growth can induce adaptive response mechanisms to environment change, such as upregulation of heat and cold shock proteins (Fayet et al., 1989; Schulz and Schumann, 1996; Zhang and Griffiths, 2003; Varcamonti et al., 2006). In our transcriptome analysis, we solely identified *hrcA* and *grpE* as genes related to heat shock, both of which were upregulated at 32°C. Detection of these important genes in fish-adapted GBS strains suggests their participation in bacterial resistance to high temperature in both aquatic and host environments. The universal stress protein (*usp*) involved in natural defense mechanisms against stress conditions was also upregulated at 32°C. It is noteworthy that cold shock is one of the few conditions which cause repression of *usp* expression in bacteria (Kvint et al., 2003). And as *usp* was induced at high temperature, we can speculate that it may induce thermal tolerance in fish-adapted GBS strains. Moreover, in GBS, *usp* has been associated with long term survival during nutrient stress (Yang et al., 2012). Therefore, the expression of *usp* in fish-adapted GBS might be essential to its survival during adaptation in aquatic environments, where nutrient deprivation and competition with other species for the same substrates occurs. And at high temperature the bacterial species have a higher affinity for nutrients, allowing the perseverance of pathogens in this stress conditions (Nedwell, 1999).

Genes involved in oxidative stress control were divergently downregulated and upregulated at 32°C. GBS strains are able to induce reactive oxygen species (ROS) production in host cells during the infectious process (Costa et al., 2016). ROS are generated by host phagocytes as part of defense mechanisms against the invasion of different microorganisms to damage essential components of bacterial cells (Zheng et al., 2017). Previous studies have suggested that GBS strains can adapt to an oxidative environment (Yamamoto et al., 2006) and persist and survive within macrophages (Shabayek and Spellerberg, 2017). In our study, the gene *trx* was upregulated at 32°C. Trx and other oxidoreductase proteins are important for maintaining the thiol state in bacterial cells through reduction of oxidized cysteine residues in cytoplasm (Ezraty et al., 2017). Higher expression of genes involved in oxidative stress at elevated temperatures coincides with the greater potential of fish-adapted GBS strain to infect and cause disease in the aquatic host. Also, the temperature increase for some fish species induces a higher production of ROS as a response to the heat stress (Banh et al., 2016). Thus, the expression of *trx* in GBS should facilitate bacterial survival in the host. On the other hand, during winter, at low temperature, the demand for anti-oxidants may be lower as the bacterium occurs more in the environment than in the host. Corroborating this statement, *noxE* (NADH oxygenase) was downregulated at 32°C. This gene assists in oxygen tolerance in GBS strains, either by decreasing the intracellular NADH/NAD+ ratio or by direct elimination of oxygen, and contributes to the bacterial infection process in the blood, liver, and brains of mice (Yamamoto et al., 2006). Moreover, *noxE* also contributes to growth of *Streptococcus* sp. in carbohydrate-limited environments (Gibson et al., 2000), indicating that the higher expression of this gene at low temperature is more related with adaptation to nutrient availability in aquatic environment than with protection against oxidative stress.

Two putative virulence genes were downregulated at 32°C: *cAMP factor* and *adhesion protein*, which is homologous to *lmb* (laminin-binding surface protein). *cAMP factor* is a protein secreted by GBS strains with pore-forming and hemolytic proprieties (Rajagopal, 2009). The high expression of this gene has been described at high temperature conditions (35°C) in fish GBS strains (Kayansamruaj et al., 2014). However, our results contradict this statement as higher expression of *cAMP factor* was detected at low temperature. *Lmb* promotes binding between GBS and host laminin, a glycoprotein of the basement membrane, and induces GBS invasion into brain tissues of human hosts (Al Safadi et al., 2010). Few fish-adapted GBS strains showed the presence of *lmb* in their genome when screened by PCR (Godoy et al., 2013). However, *lmb* has not yet been studied in terms of its biological functions in these strains.

### Changes in the proteome are modulated by temperature

In our proteomic analysis, we observed that temperature could influence metabolic pathway protein abundance (Figure 3B). Of proteins downregulated at 32°C, we identified proteins involved in carbohydrate metabolism including DexB and glycerol uptake permease. DexB (Glucan 1,6-alpha-glucoside) participates in the starch and sucrose metabolic pathway and releases glucose from the non-reducing terminus of alpha-1,6-linked dextran or isomaltosaccharides (Whiting et al., 1993). The released free glucose units may be used by fish-adapted GBS strains in the gluconeogenesis pathway, even during adaptation to low temperature when glucose affinity is decreased (Nedwell, 1999). Glycerol uptake permease is an aquaporin responsible for conducting water and small hydrophilic solutes, particularly glycerol, into the bacterial cell (Lu et al., 2003). Glycerol uptake at low temperatures can also contribute to obtain an alternative energy source for bacterial growth in this condition.

Proteins involved in inorganic ion transport and metabolism, such as phosphate ABC transporters (PtsAB), nickel ABC transporters, and cobalt ABC transporters were downregulated at 32°C. Inorganic ions, like iron, cobalt, nickel, copper are required by bacteria to carry out biological processes and are important enzymatic cofactors acquired mainly from the environment, that contribute to the biological activities of many bacterial proteins (Hohle et al., 2011; Schreur et al., 2011). Expression of some of these proteins has been previously shown to be upregulated at high temperatures (Mereghetti et al., 2008), contradicting our findings. Some genes involved in inorganic ion metabolism are absent or inactive in fish-adapted GBS strains, affecting ion exchange and reducing the bacterial ability to maintain homeostasis in unfavorable environmental conditions (Rosinski-Chupin et al., 2013). However, for these strains, proteins involved in inorganic and metallic ion metabolism were identified in a label-free pan-proteomics analysis and were considered important for the growth and survival of pathogens in aquatic environment (unpublished data). PstAB are responsible for bacterial growth in environment with limited inorganic phosphate concentration and by adhesion of *S. mutans* in abiotic surfaces (Ferreira et al., 2016). Nickel uptake by ABC transporters is essential for maintaining a neutral bacterial cytosolic pH and to allow colonization of *S. aureus* and *Helicobacter pylori* in human cells (Tanaka et al., 2018). Cobalt is important for bacterial energy metabolism and anabolism, being found in the corrin ring of coenzyme B_12_. This metallic ion competes with iron in several metabolic pathways and adaptive changes in response to environmental cobalt concentration (Majtan et al., 2011). Therefore, the differential protein abundance of this functional category at low temperature may contribute to the bacterial viability in an environment limited in essential metal ions.

A change in regulatory direction was observed in proteins related to purine metabolism and oligopeptide uptake, when compared to transcriptome data. In the proteome, OppC protein was downregulated at 32°C, whereas PurH, PurK, and PurM were upregulated at 32°C. This change may have occurred because the samples were collected at the same time point. However, transcripts and proteins have different half-lives, and changes in transcript expression only affect protein levels after a certain temporal delay, as protein synthesis takes time (Liu et al., 2016).

In our study, RNA helicase was identified as downregulated at 32°C with its abundance altered by 2.08-fold. RNA helicase is a cold shock protein involved in mRNA processing, transport, or degradation, ribosome biogenesis, and translation initiation during cold acclimation (Phadtare and Severinov, 2010). Similarly, the RNA helicase *deaD* gene was upregulated at 30°C relative to 40°C in a previous study on a GBS strain during the stationary phase of bacterial growth (Mereghetti et al., 2008). In our proteomic dataset, GroS and ClpL (both heat shock proteins) were downregulated at 32°C. Clp ATPases, like ClpL, are involved in the folding, assembly, and proteolysis of proteins (Nair et al., 2003), whereas GroS prevents inactivation of cellular proteins and assists in the degradation of non-repairable denatured proteins, which may accumulate during bacterial growth in normal or stress conditions (Yura et al., 1993). A previous study showed that ClpL protein synthesis is enhanced in *Streptococcus thermophilus* cells stored at high and low temperature conditions (Varcamonti et al., 2006). In GBS cells, however, there was no differential expression of GroS and ClpL during growth at 30 and 40°C, suggesting that proteins are always required during bacterial growth, regardless of growth temperature (Mereghetti et al., 2008). Therefore, our results indicate that fish-adapted GBS presented the same phenomenon of expressing heat shock proteins at both temperatures. However, higher expression of heat and cold shock proteins at low temperature can represent an important mechanism of protection and homeostasis in this condition.

Proteins involved in oxidative stress control were upregulated at 32°C. Alkyl hydroperoxide reductase subunit C (AhpC), Trx and flavodoxin showed a change in abundance level of 2.07-, 6.2- and 3.4-fold, respectively. AhpC is an important protein involved in defense against different substrates as H_2_O_2_ and organic peroxides, participating of detoxification of ROS formed in bacterial cells or derived from the host. This protein is responsible for the induction and maintenance of the viable but non-culturable state in *Vibrio parahaemolyticus* (Wang et al., 2013). Similar to Trx (described above), flavodoxin defends against ROS and contributes to *Pseudomonas aeruginosa* survival in macrophagic cells and in *Drosophila melanogaster* (Moyano et al., 2014). Thus, AhpC and flavodoxin might also contribute to the survival of fish-adapted GBS strains in fish cells during the infection process. Notably, in Nile tilapia, the respiratory burst activity and phagocytic activity of macrophages decreases with both reduction in temperature and exposure time to the aquatic environment (Qiang et al., 2018). In this manner, a higher macrophage activity at high temperature could decrease the sensitivity of fish to pathogens. However, in this condition, fish-adapted GBS showed higher abundance of proteins involved in oxidative stress control, which leads us to speculate that at 32°C, the GBS isolate required greater protection against ROS to survive under environment or during infection in the aquatic host.

Four proteins related to bacterial pathogenicity showed differential protein abundance between 22 and 32°C (Table 2). Reticulocyte binding protein, homologous to C5a peptidase (ScpB), was downregulated at 32°C, and showed a change in abundance level of 20.8-fold. This protein is a serine protease involved in adhesion and host immune evasion (Rajagopal, 2009). On the other hand, IagA (4.1-fold), CpsB (2-fold), and cAMP factor (2.7-fold) were upregulated at 32°C. In human infections by GBS, IagA contributes to blood-brain barrier invasion (Doran et al., 2005), whereas CpsB, a protein related to capsule synthesis, protects the bacterium though prevention of complement deposition (factor C3b) and opsonophagocytosis (Glaser et al., 2002). cAMP factor also switched its direction of regulation compared to the transcriptome analysis, being upregulated at 32°C in proteome analysis. As previously reported for the *lmb* gene, neither of these virulence factors have been their rules evaluated in fish infection models. However, identification of reticulocyte binding protein, IagA, CpsB, and cAMP factor indicates that these proteins may contribute to the adhesion, dissemination, and survival of GBS in fish tissues at both temperature conditions, with a pathogenesis landscape similar to human GBS infections.

### Temperature and pathogenicity

It is known that GBS can be isolated from diseased fish at low temperatures (Chideroli et al., 2017). It has been clearly demonstrated that fish can become infected under these conditions, and that GBS strains should therefore use virulence factors such as reticulocyte binding protein and *lmb*, under these conditions to cause disease in the aquatic host. In field conditions, when tilapia are cultured in water at high temperatures, the GBS load in the tissue of infected fish can increase, causing extensive tissue damage with a massive inflammatory response, thus increasing the expression of several virulence genes, allowing GBS to transcend the host-defense mechanism, and increasing the incidence of death in fish (Kayansamruaj et al., 2014). An experimental infection assay with GBS in Nile tilapia cultured at 32 and 22°C (the same temperatures used in our *in vitro* assay) showed that all fish died after three days and in the second week post-challenge, respectively (Wang et al., 2016). Another experimental infection showed cumulative mortality rates of 70, 50, and 0% in tilapia adapted to water temperatures of 30, 25, or 20°C, respectively (Zhao et al., 2015). In Brazil, an experimental challenge with GBS performed in tilapia acclimated to water at 22 and 31°C showed mortality rates of 10 and 80%, respectively (Chideroli et al., 2017). In our study, we expected to find several proteins related to pathogen-host interaction being highly abundant at 32°C compared to 22°C, because there are higher mortality rates at this temperature.

For several bacterial pathogens of mammals, the main trigger for the expression of virulence factors in pathogenic bacteria is temperature (Guijarro et al., 2015). At high temperature (37°C), *Shigella* spp. (Maurelli et al., 1984), *Bordetella pertussis* (Rappuoli et al., 1992), *Yersinia pestis* (Karlyshev et al., 1992), and *E. coli* (Falconi et al., 1998) showed virulence gene expression to be stimulated by environmental factors, becoming virulent for the mammalian host. On the other hand, in fish-pathogenic bacteria, upregulation of virulence factors is commonly verified at low temperature, as observed in *Yersinia ruckeri* (18 vs. 28°C; Méndez et al., 2009), *Flavobacterium psychrophilum* (12 vs. 18°C; Gómez et al., 2012), *L. garvieae* (18 vs. 37°C; Aguado-Urda et al., 2013), *Aeromonas hydrophila* (25 vs. 37°C; Yu et al., 2007), and *Edwardsiella tarda* (25 vs. 37°C; Srinivasa Rao et al., 2004). In our study, genes/proteins involved in the virulence of GBS were detected at both temperature conditions, indicating that their expression is not totally temperature-dependent and the bacterial virulence machinery always active. Non-differentiation of some virulence factors expression may be related to the fact that fish GBS is frequently exposed to the environment (water and fish tissues), conferring an adaptive phenotypic plasticity necessary to maintain infectivity in a broad range of body temperatures. Nevertheless, genes/proteins that play a key role in pathogenicity were detected with differential abundance among the temperatures. Although the fish-adapted GBS strains had evolved in divergent branch from mammalian GBS isolates (Barony et al., 2017), several genes are shared among piscine and mammalian strains (Rosinski-Chupin et al., 2013; Kawasaki et al., 2018), including those identified as differentially expressed in our study. Therefore, upregulation at 32°C of proteins IagA, CpsB and cAMP factor may be important for the onset of streptococosis in tilapia during the warmer months. However, in Brazil, GBS have been considered endemic in tilapia farms, being detected in diseased and carrier fish in field condition throughout the year, even during the winter (Delphino et al., 2019). In this way, virulence factors detected in the proteome of GBS in low temperature may contribute to the maintenance of the bacteria in carrier (apparently healthy) fish during the cold months.

### Low correlation between transcriptomic and proteomic datasets

A weak relationship between transcriptomic and proteomic profiles was observed in our analysis. Correspondence among these datasets has been reported as low for different bacterial pathogens (Dressaire et al., 2010; Margalef-Català et al., 2016), including bacteria of the *Streptococcus* genus (Ahn et al., 2017), due to different regulatory mechanisms involved in gene expression and protein synthesis (Haider and Pal, 2013) and technical limitations among the approaches used (Lundberg et al., 2010). Among the physiological mechanisms involved in this low correlation between transcriptome and proteome, half-lives of mRNAs and proteins, and post-transcriptional machinery such as translation and protein degradation and modification have been described (Haider and Pal, 2013; Ahn et al., 2017). The half-life of mRNA in bacteria is short, varying from seconds to over an hour (Laalami et al., 2014) and is much shorter than their corresponding proteins, which have stable half-lives (>20 h; Deller et al., 2016). In addition, regulatory factors may be responsible for the low correlation between transcriptomic and proteomic datasets, especially of protein nature, where some proteins were not detected in our analysis due of limitations of detection capacity of the method employed.

## Conclusions

The present study is the first to characterize the functional genome of fish-adapted GBS strains at transcriptomic and proteomic levels under different temperature conditions. The data indicate that the transcriptome and proteome of fish-adapted GBS are modulated by temperature. A greater variation in the expression of the functional genome was observed in transcriptome analysis, especially at 32°C. Our comparison analysis demonstrated that fish-adapted GBS regulates the differential expression of transcripts and proteins involved in metabolism and adaptation to aquatic environment and to the fish host at low and high temperatures. Temperature was also able to influence the differential expression of some virulence factors at both low and high temperatures, which indicates the ability of colonization of the aquatic host in both conditions. The results obtained in this study open prospects for future investigations on the expression trends of genes and proteins enabling bacterial adaptation to aquatic environments or their survival and growth inside the fish host.

## Author contributions

GT performed microbiological analyses and sample preparation for transcriptomic and proteomic analyses. GT and AC conducted the transcriptomic analysis. GT and CR conducted the proteomic analysis. GT and FP performed bioinformatics analysis of data. GT wrote the manuscript. FP, CL, and VA contributed substantially to data interpretation and revisions. HF coordinated all analyses of the project and critically reviewed the manuscript. All authors read and approved the final manuscript.

### Conflict of interest statement

The authors declare that the research was conducted in the absence of any commercial or financial relationships that could be construed as a potential conflict of interest.

## Supplementary material

Supplementary Table 1 Genes selected and primers used for validation of microarray results.

Supplementary Table 2 Complete list of transcripts identified by microarray analysis during GBS growth at 22°C and 32°C.

Supplementary Table 3 Complete list of proteins identified by LC-HDMS^E^ analysis during GBS growth at 22°C and 32°C.

Supplementary Table 4 List of ten most and least expressed proteins identified by LC-HDMS^E^ in GBS growth at 22°C and 32°C.

Supplementary Figure 1 Quality assessment of the biological replicates used in transcriptome analysis. **(A)** Distribution of the intensities evaluated by box plot; **(B)** Correlation analysis matrix. **(C)** PCA plot, red circles represent samples grown at 32°C, whereas blue circles represent samples grown at 22°C.

Supplementary Figure 2 Interaction networks of DEGs **(A)** and DPAs **(B)** identified in strain SA53 between the two temperatures. Red circles represent proteins upregulated at 32°C, whereas blue circles represent proteins downregulated at 32°C. (1) PTS systems; (2) ABC transport system; (3) Ascorbate and aldarate metabolism; (4) Purine metabolism; (5) metabolic pathways.

Supplementary Figure 3 Quality assessment of biological replicates used in proteome analysis. **(A)** Normal distribution of 10 ppm error in the total identified peptides; **(B)** repeat rate indicating the number of times that an identified protein appears in the replicates: 3 of 3 (blue) and 2 of 3 (red). **(C)** PCA plot, red circles represent samples grown at 32°C, whereas blue circles represent samples grown at 22°C.
