# Supplementary material for: Transcriptome and Proteome of Fish-Pathogenic Streptococcus agalactiae Are Modulated by Temperature
Source: Front Microbiol. 2018 Nov 2;9:2639. doi: 10.3389/fmicb.2018.02639 (PMC6224512; doi:10.3389/fmicb.2018.02639)
Supplement: Supplementary Table 1 — Genes selected and primers used for validation of microarray results. [file Table_1.DOCX]

| **Primer** | **Product** | **Sequence (5’-3’)** | **Product size (bp)** | **Microarray^a^** | **qRT-PCR^b^** |
| --- | --- | --- | --- | --- | --- |
| SagaV-20_0110F | Pyridine nucleotide-disulphide oxidoreductase family protein | GAATTAGGGAGCAAAGTGACAG | 100 | 2.11 | 1.09 |
| SagaV-20_0110R |  | TCTCTTCTAAATAATCTTGAGCCATCTCT |  |  |  |
| SagaV-20_1663F | Methylenetetrahydrofolate reductase | TTGGGCATGGAATTATTAGGTTTT | 119 | -4.84 | -0.54 |
| SagaV-20_1663R |  | GAAAGGAGTTTAAAGGAAGTCACATCA |  |  |  |
| SagaV-53_0507F | Acetoin reductase | GCAATCAATGTCGGAGGAACTAT | 80 | 2.49 | 2.43 |
| SagaV-53_0507R |  | TTTCCGCCATGACCTAATTCTC |  |  |  |
| SagaV-53_1711F | 5-methyltetrahydropteroyltriglutamate | TGAAGCAGCCCTTCGAGAAG | 75 | -4.04 | -0.57 |
| SagaV-53_1711R |  | GCCGCAACAGCATCATCTAA |  |  |  |
| SagaV-gyrAF | DNA gyrase subunit A | GCAGTGAACGTGAACCTCTTGTT | 99 | Reference gene | |
| SagaV-gyrAR |  | GGTGGAATATTTGTTGCCATACCT |  |  |  |
| SagaV-recAF | Protein RecA | AAAACAACGGTTGCCCTTCA | 99 | Reference gene | |
| SagaV-recAR |  | CAGCATAGGCTGGGTCAAGAG |  |  |  |

a Genes with log2 ≥ 1 = up-regulated; or ≤ -1 = down-regulated

b ∆∆Ct value
