# Supplementary figures and images for: Transcriptome and Proteome of Fish-Pathogenic Streptococcus agalactiae Are Modulated by Temperature
Source: Front Microbiol. 2018 Nov 2;9:2639. doi: 10.3389/fmicb.2018.02639 (PMC6224512; doi:10.3389/fmicb.2018.02639)

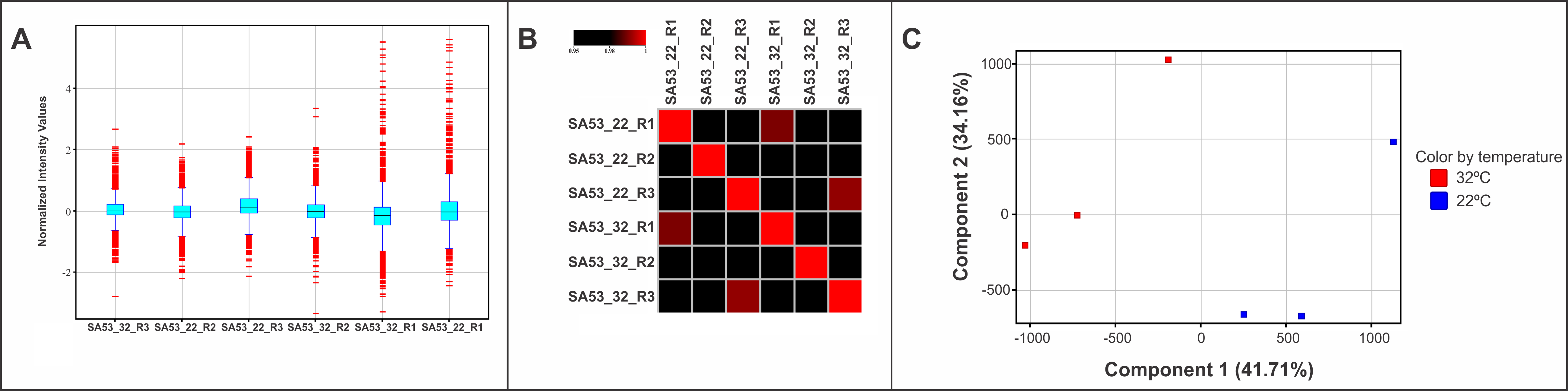

Supplement: Supplementary Figure 1 — Quality assessment of the biological replicates used in transcriptome analysis. (A) Distribution of the intensities evaluated by box plot; (B) Correlation analysis matrix. (C) PCA plot, red circles represent samples grown at 32°C, whereas blue circles represent samples grown at 22°C. [file Image_1.TIF]

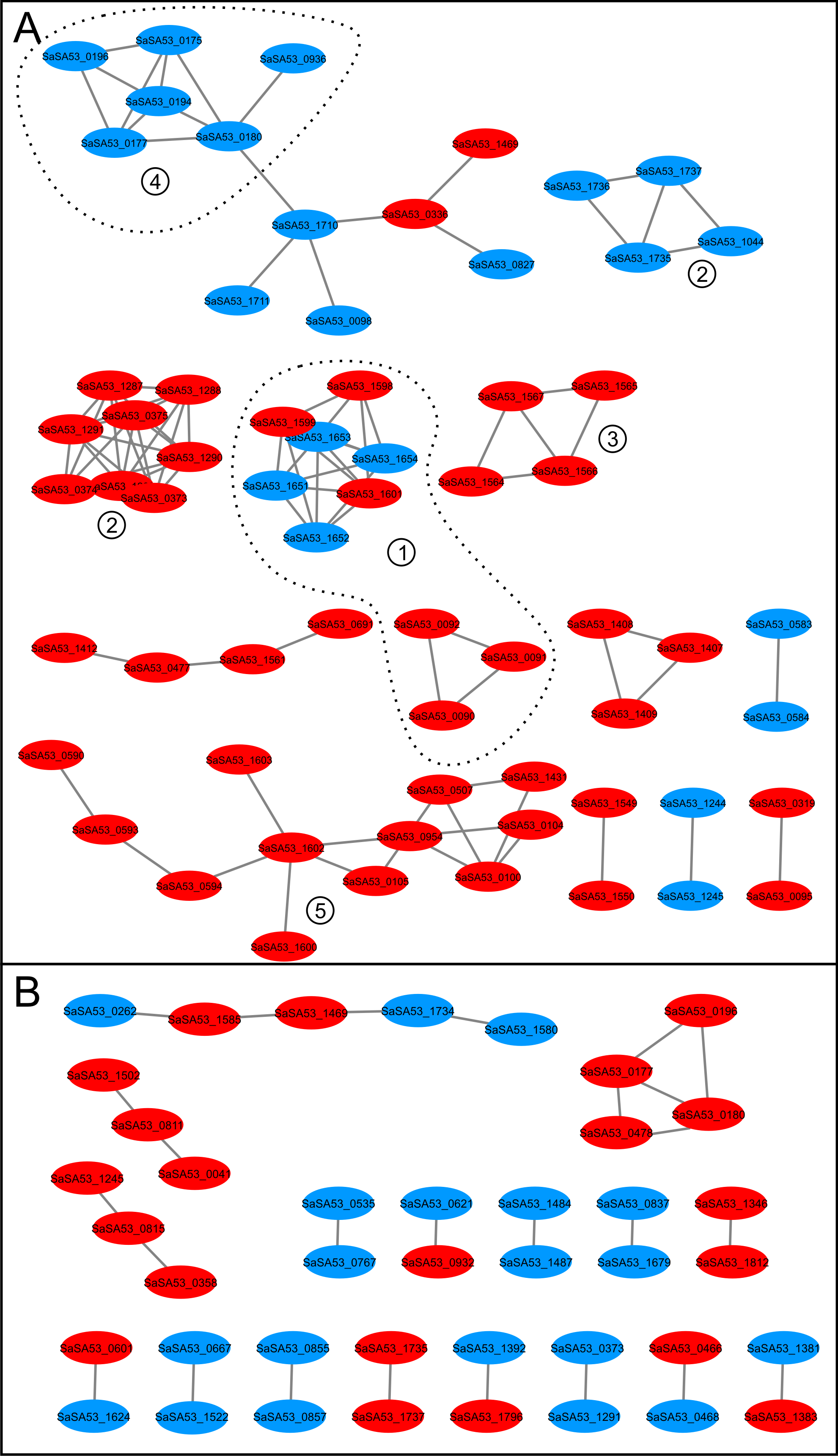

Supplement: Supplementary Figure 2 — Interaction networks of DEGs (A) and DPAs (B) identified in strain SA53 between the two temperatures. Red circles represent proteins upregulated at 32°C, whereas blue circles represent proteins downregulated at 32°C. (1) PTS systems; (2) ABC transport system; (3) Ascorbate and aldarate metabolism; (4) Purine metabolism; (5) metabolic pathways. [file Image_2.TIF]

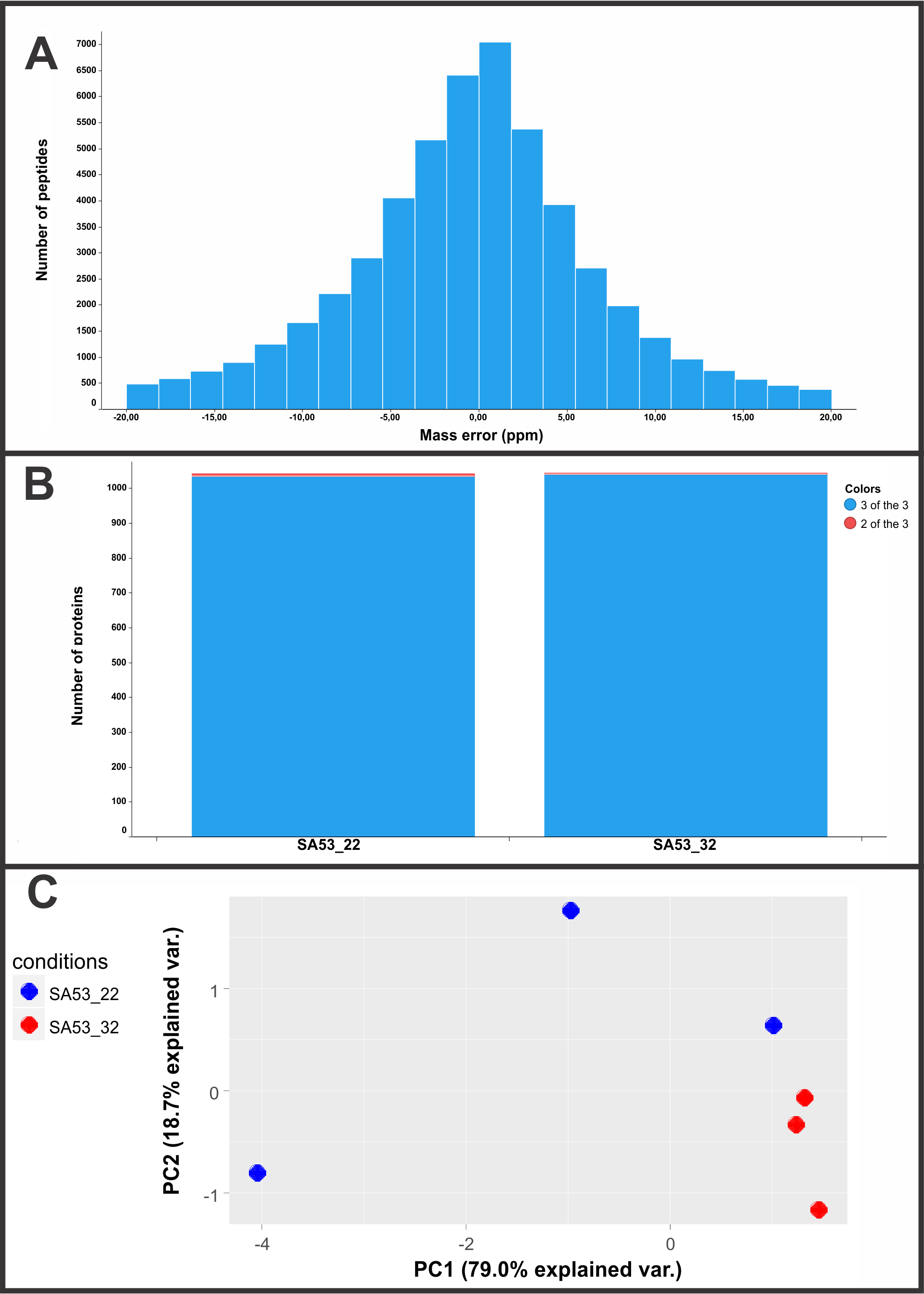

Supplement: Supplementary Figure 3 — Quality assessment of biological replicates used in proteome analysis. (A) Normal distribution of 10 ppm error in the total identified peptides; (B) repeat rate indicating the number of times that an identified protein appears in the replicates: 3 of 3 (blue) and 2 of 3 (red). (C) PCA plot, red circles represent samples grown at 32°C, whereas blue circles represent samples grown at 22°C. [file Image_3.TIF]
